# Decreased Right Temporal Activation and Increased Interhemispheric Connectivity in Response to Speech in Preterm Infants at Term-Equivalent Age

**DOI:** 10.3389/fpsyg.2013.00094

**Published:** 2013-03-01

**Authors:** Nozomi Naoi, Yutaka Fuchino, Minoru Shibata, Fusako Niwa, Masahiko Kawai, Yukuo Konishi, Kazuo Okanoya, Masako Myowa-Yamakoshi

**Affiliations:** ^1^Okanoya Emotional Information Project, The Exploratory Research for Advanced Technology, Japan Agency of Science and TechnologySaitama, Japan; ^2^Graduate School of Education, Kyoto UniversityKyoto, Japan; ^3^Department of Pediatrics, Kyoto University HospitalKyoto, Japan; ^4^Center for Baby Science, Doshisha UniversityKyoto, Japan; ^5^Department of Life Sciences, Graduate School of Arts and Sciences, The University of TokyoTokyo, Japan

**Keywords:** infant-directed speech, adult-directed speech, full-term neonates, preterm infants, near-infrared spectroscopy

## Abstract

Preterm infants are at increased risk of language-related problems later in life; however, few studies have examined the effects of preterm birth on cerebral responses to speech at very early developmental stages. This study examined cerebral activation and functional connectivity in response to infant-directed speech (IDS) and adult-directed speech (ADS) in full-term neonates and preterm infants at term-equivalent age using 94-channel near-infrared spectroscopy. The results showed that compared with ADS, IDS increased activity in larger brain areas such as the bilateral frontotemporal, temporal, and temporoparietal regions, both in full-term and preterm infants. Preterm infants exhibited decreased activity in response to speech stimuli in the right temporal region compared with full-term infants, although the significance was low. Moreover, preterm infants exhibited increased interhemispheric connectivity compared with full-term controls, especially in the temporal and temporoparietal regions. These differences suggest that preterm infants may follow different developmental trajectories from those born at term owing to differences in intrauterine and extrauterine development.

## Introduction

The incidence of preterm birth before 37 weeks gestation has increased worldwide in recent years (Tracy et al., 2007; Gray et al., 2008; Vanderweele et al., 2012). In Japan, it has increased from 4.1% of all live births in 1980 to 5.7% in 2010 (Japan Ministry of Health, Labour and Welfare, 2011). Medical advancements in neonatology have significantly increased the survival rate of preterm infants (Larroque et al., 2004; Fanaroff et al., 2007; Gaddlin, 2011; Kono et al., 2011). Recent reports have indicated an approximate survival rate of 90% in Japan for preterm infants with birth weights below 1500 g (Kusuda et al., 2006; Itabashi et al., 2009; Kono et al., 2011).

Despite these improvements in survival of preterm infants, recent follow-up studies have suggested that preterm children are at risk of problems related to cognitive, language, and emotional development (Aylward, 2002; Jansson-Verkasalo et al., 2004b; Salt and Redshaw, 2006; Luu et al., 2009a,b). With respect to language problems, studies have shown that preterm preschoolers have significantly lower receptive vocabularies and expressive language skills, such as naming, even without obvious brain injuries (Aram et al., 1991; Luoma et al., 1998). While some studies have suggested that receptive language skills in preterm children improve by adolescence, deficits in more complex language skills, such as phonological awareness and phonemic decoding, which are important for academic skills such as reading, persist into adolescence, and early adulthood (Aylward, 2002; Aarnoudse-Moens et al., 2009; Luu et al., 2009a, 2011).

Advances in neuroimaging techniques have provided important information about the effect of preterm birth on functional cerebral development. Using magnetoencephalography (MEG) during an orthographic–phonological reading task, Frye et al. (2009) examined neuromagnetic responses of adolescents born preterm with low and high medical risks (primarily bronchopulmonary dysplasia) but without obvious brain injuries sustained during the neonatal period. The results demonstrated that preterm adolescents born under high-risk conditions exhibited increased activation in the left Broca’s and prefrontal areas compared with preterm adolescents born under low-risk conditions and those born full-term. Frye et al. (2010) also reported that during an auditory phonological task, preterm adolescents born under high-risk conditions demonstrated decreased activation in the right superior temporal gyrus and overactivation in the left Broca’s and prefrontal areas compared with preterm adolescents born under low-risk conditions and those born full-term. During both orthographic and auditory phonological tasks, activation of the left Broca’s and prefrontal areas, which is associated with better phonological skills, was observed only in preterm adolescents born under high-risk conditions. The authors suggested that left Broca’s and prefrontal activation in preterm adolescents born under high-risk conditions might reflect compensatory recruitment of alternative neural mechanisms for language processing.

Recent studies on brain functional connectivity have also demonstrated that preterm children and adolescents exhibit alternative neural networks for processing language compared with full-term controls (Gozzo et al., 2009; Schafer et al., 2009; Myers et al., 2010). Functional connectivity is defined as “temporal correlations of activity between spatially remote cortical regions” (Friston et al., 1996; Biswal et al., 1997; Fox and Raichle, 2007; Fair et al., 2008; Sasai et al., 2011) and is currently assessed by blood oxygen level-dependent signals using functional magnetic resonance imaging (fMRI; Friston et al., 1996; Biswal et al., 1997; Fox and Raichle, 2007; Fair et al., 2008; Sasai et al., 2011) and hemoglobin oxygenation signals using near-infrared spectroscopy (NIRS; White et al., 2009; Homae et al., 2010; Chaudhary et al., 2011; Medvedev et al., 2011; Sasai et al., 2011). Recently, functional connectivity examinations have been performed to determine the effect of preterm birth on cerebral cortex development not only during the resting state (Doria et al., 2010; Smyser et al., 2010) but also during language-related tasks (Gozzo et al., 2009; Schafer et al., 2009; Myers et al., 2010). Using fMRI, Gozzo et al. (2009) demonstrated that during a passive auditory listening task, preterm children at school age (about 9 years) exhibited increased connectivity between Wernicke’s area in the left superior temporal gyrus and the right inferior frontal gyrus (Broca’s area homolog) and both the left and right supramarginal gyri in the parietal region, compared with full-term controls. At about 12 years, during a semantic association task, preterm children exhibited increased connectivity between Wernicke’s area and the sensory motor areas compared with full-term controls (Schafer et al., 2009). Furthermore, functional connectivity between Wernicke’s area and the right supramarginal gyrus was increased in preterm adolescents (at about 16 years) during a match/mismatch judgment task of pictures and words that presented acoustically and/or visually (Myers et al., 2010).

Taken together, results of recent brain activation and functional connectivity studies converge to suggest development of alternative neural systems for processing language in preterm children and adolescents. Although clear relationships have been demonstrated between preterm birth and later language-related difficulties during childhood and adolescence, little is known about when or how these problems arise in the course of development.

Peña et al. (2010) examined the gamma-band response in younger infants and the event-related potential (ERP) in response to the infants’ native language, a rhythmically similar non-native language, and a rhythmically distant non-native language in 3- and 6-month-old full-term infants and 6- and 9-month-old preterm infants (3 and 6 months corrected age). There was an increased gamma-band power in response to the native language compared with the rhythmically similar non-native language in 6-month-old full-term infants and preterm infants at 9 months postnatal age (PNA; almost 6 months term-corrected age). These results suggest that neural maturation appears to constrain the effects of postnatal experience on speech discrimination in preterm infants. Furthermore, the relationship between the behavioral and neural response to speech signals was examined in children born preterm. Using an ERP component called mismatch negativity (MMN) as an indicator of auditory discrimination, Jansson-Verkasalo et al. (2003) examined the relationship between development of object naming ability and auditory processing in preterm preschoolers with very low birth weight (VLBW; less than 1500 g) at around 4 years of age. MMN amplitude for consonant change was significantly smaller in preterm children compared to with full-term controls. In addition, MMN amplitude varied as a function of children’s object naming ability in that weaker naming by preterm children was associated with diminished MMN amplitudes. In addition, absence of MMN for consonant change at 4 years was predictive of naming ability deficits at 6 years (Jansson-Verkasalo et al., 2004a). Furthermore, Jansson-Verkasalo et al. (2010) compared MMN for vowel changes in the infants’ native and non-native languages in preterm and full-term infants at corrected ages of 6 and 12 months. Both preterm and full-term infants at 6 months exhibited MMN for both native and non-native vowel changes; however, MMN for non-native vowel changes diminished at 12 months only in full-term infants. In contrast, MMN for non-native vowel changes persisted in preterm infants at 12 months. Furthermore, the absence of MMN attenuation at 12 months in preterm infants was predictive of deficits in expressive language ability at 2 years. Thus, difficulties in specialization to native language early in development might be associated with later language problems in preterm children.

Only few studies have investigated the cerebral basis of speech processing in preterm and full-term infants during the earliest postnatal developmental stage. Preterm infants are born earlier than full-term infants so they have less intrauterine maturation at birth.

Previous MRI studies have suggested that intrauterine rather than extrauterine development appears to have an adequate impact on brain structure maturation in preterm infants (for a recent review, see Mento and Bisiacchi, 2012). When infants were scanned at term-equivalent age, preterm infants were found to exhibit reduced cortical sulcation and reduced white and gray matter volume compared with full-term controls even when there were no obvious brain injuries (Peterson et al., 2003; Inder et al., 2005). In addition, diffusion tensor imaging (DTI) studies have detected alterations in the cerebral white matter microstructure of preterm infants compared with full-term infants (Huppi et al., 1998; Aeby et al., 2009; Skiold et al., 2010). These cerebral structural alterations are known to be significantly correlated to the gestational age (GA) at birth and these can predict adverse developmental outcomes (Peterson et al., 2003; Inder et al., 2005; Kapellou et al., 2006; Woodward et al., 2006).

Similarly, previous functional studies have also demonstrated a strong dependence of functional brain maturation on intrauterine rather than extrauterine development in preterm infants. In full-term neonates, brain processing of speech stimuli varies with postnatal auditory experiences, and more exposure to familiar stimuli results in larger ERP amplitudes (Cheour et al., 2002; deRegnier et al., 2002). On the other hand, Therien et al. (2004) reported that preterm infants measured at term-equivalent age exhibited different polarity and scalp distribution of ERP for speech sounds (syllable changes) compared with term infants. In addition, Key et al. (2012) examined the effect of gestational and PNA on speech sound perception in preterm and full-term infants treated in the neonatal intensive care unit (NICU) using ERP. The results showed that increasing PNA was associated with greater ERP amplitude differences for consonant changes only in infants born at or after 30 weeks gestation, while in preterm infants born before 30 weeks gestation, PNA was not associated with ERP amplitude differences despite increased postnatal experience.

Although the results of these ERP studies provide some evidence that alterations in neural systems for processing speech occur in preterm infants even at term-equivalent age, these findings provide limited information about spatial localization of speech processing in preterm infants. In the present study, we examined cerebral responses of full-term neonates and preterm infants to infant-directed speech (IDS) and adult-directed speech (ADS) by simultaneously measuring activations in the frontal, temporal, parietal, and occipital regions using multichannel NIRS. If development of neonate brain response to speech is determined by maturation, we would expect to find similar activation patterns in preterm and term infants with equal postmenstrual age (PMA). On the other hand, if neonate brain response to speech is affected by preterm birth, we would expect to find different activation patterns in preterm and full-term infants. We addressed these questions by examining not only cerebral activation in response to speech stimuli but also functional connectivity of the different brain regions involved in speech processing.

## Materials and Methods

### Participants

Twenty-seven preterm infants and 29 full-term newborn infants participated in this study. Two preterm infants and four full-term infants were excluded from the final statistical analysis because of excessive motion artifacts (preterm = 2 and full-term = 1) or hair obstruction (full-term = 3). The final sample included 25 preterm infants (11 girls) and 25 full-term infants (10 girls). Demographic information for the preterm and full-term infant groups is summarized in Table 1.

**Table 1:** **Demographic information for preterm and full-term infant groups**.

	Preterm (*n* = 25)		Full-term (*n* =25)		*p*-Value
	Mean	SD	Mean	SD	
**AT BIRTH**					
Gestational age (days)	218.3	25.8	270.6	9.0	<10^−9^
Birth weight (g)	1488.9	567.8	2927.9	439.6	<10^−12^
Apgar 5 min	8.1	1.3	9.2	0.4	<10^−3^
**AT NIRS MEASUREMENT**					
Postnatal age (days)	56.7	31.0	4.7	2.0	<10^−7^
Postmenstrul age (days)	275.4	9.8	275.3	9.0	0.98
Head circumference (cm)	33.9	1.7	34.1	2.0	0.71
Female	11/20		10/20		

Preterm infants born before 37 complete weeks of gestation and with a birth weight below 2500 g were included in the study. Among preterm infants, 11 were born with a birth weight below 1500 g (VLBW) and 7 below 1000 g (extremely low birth weight, ELBW). Children with severe neurologic complications such as brain lesions including periventricular leukomalacia (PVL) and intraventricular hemorrhage (IVH) and those with impaired hearing or vision were not included in the study.

In preterm infants, the mean GA at birth was 218.3 days [standard deviation (SD) = 25.8] and the mean birth weight was 1488.9 g (SD = 567.8). The mean Apgar score 5 min after delivery was 8.1 (SD = 1.3). Preterm infants were measured using multichannel NIRS at term PMA. The mean PNA at NIRS measurement was 56.7 days (SD = 31.0) and mean PMA at NIRS measurement was 275.4 days (SD = 1.4). In addition, mean head circumference at NIRS measurement was 33.9 cm (SD = 1.7).

In full-term control infants, the mean GA at birth was 270.6 days (SD = 9.0) and the mean birth weight was 2927.9 g (SD = 439.6). The mean Apgar score 5 min after delivery was 9.2 (SD = 0.4). The mean PNA at NIRS measurement was 4.7 days (SD = 2.0) and mean PMA at NIRS measurement was 275.3 days (SD = 9.0). In addition, mean head circumference at NIRS measurement was 34.1 cm (SD = 2.0). None of the full-term infants exhibited signs of neurological disorders or hearing or visual impairment.

The preterm infant group had significantly lower GA at birth [*t*(48) = −9.58, *p* < 10^−9^], lower birth weight [*t*(48) = −10.02, *p* < 10^−12^], lower Apgar score 5 min after delivery [*t*(48) = −4.02, *p* < 10^−3^], and higher PNA [*t*(48) = 8.30, *p* < 10^−7^] at the time of measurement. On the other hand, there was no significant difference between preterm and full-term infants in PMA (*t* = 0.03, *p* = 0.98) and head circumference at NIRS measurement (*t* = −0.38, *p* = 0.71).

All infants were in a state of natural sleep during NIRS measurement. The study was conducted with the approval of the ethics committee of Kyoto University Hospital (No. E581). Written informed consent was obtained from parents before infants were enrolled in the study.

### Stimuli

In this study, we presented speech and non-speech auditory stimuli. The speech stimuli consisted of six Japanese sentences, which were read by four Japanese female speakers as if they were speaking to their own babies (IDS) or to a female adult researcher (ADS). The recordings were made in a sound-attenuated room using a Marantz PMD 670 digital recorder and a Shure KSM27/SL microphone at a sampling rate of 44.1 kHz with 16-bit quantization. The procedures used for speech stimuli recording were similar to those reported by Naoi et al. (2012).

The mean duration of a sentence was 1.8 s (SD = 0.1 s) for the ADS samples and 2.3 s (SD = 0.1 s) for the IDS samples. In addition, the mean intersentence pause duration was 0.7 s (SD = 0.1 s) for the ADS samples and 1.2 s (SD = 0.4 s) for the IDS samples. The mean sentence duration and intersentence pause duration were significantly longer for the IDS samples than for the ADS samples [paired *t*(23) = 6.86, *p* < 0.0001, paired *t*(19) = 5.47, *p* < 0.0001, respectively]. Previous studies have shown that IDS utterances are slower than ADS utterances, even when speaking the same sentences (Stern et al., 1983; Naoi et al., 2012). Six Japanese sentences used as IDS speech stimuli was approximately 5 s longer than those used as ADS speech stimuli. Our priority was to maintain the natural characteristics of IDS, so we did not adjust the durations of IDS stimuli to equalize the lengths of IDS and ADS. Instead, we removed individual differences by editing all IDS stimuli to produce sequences of 20 s, while ADS stimuli were edited into sequences of 15 s by adjusting the pause durations.

The mean pitch of the six sentences was significantly higher in IDS stimuli than in ADS stimuli [paired *t*(23) = 9.42, *p* < 10^−5^]. The mean pitch range, which was calculated by subtracting the minimum pitch from the maximum pitch, was significantly greater for IDS stimuli than ADS stimuli [paired *t*(23) = 7.44, *p* < 10^−5^].

The non-speech control stimulus (20 s duration) was constructed using pink noise [power spectral density inversely proportional to the frequency (1/*f*)]. The mean intensities of the IDS, ADS, and pink noise samples had equal root mean square (RMS) amplitudes.

### NIRS recordings

The multichannel NIRS system (ETG-7000, Hitachi Medical Co., Tokyo, Japan) was used to measure changes in relative concentrations of oxygenated (oxy-Hb) and deoxygenated hemoglobin (deoxy-Hb). Two near-infrared lights with wavelengths of approximately 785 and 830 nm were used in this system. The sampling rate was 10 Hz. NIRS probes described by Homae et al. (2010); Taga et al. (2011), and Watanabe et al. (2012) were used.

**Figure 1:**
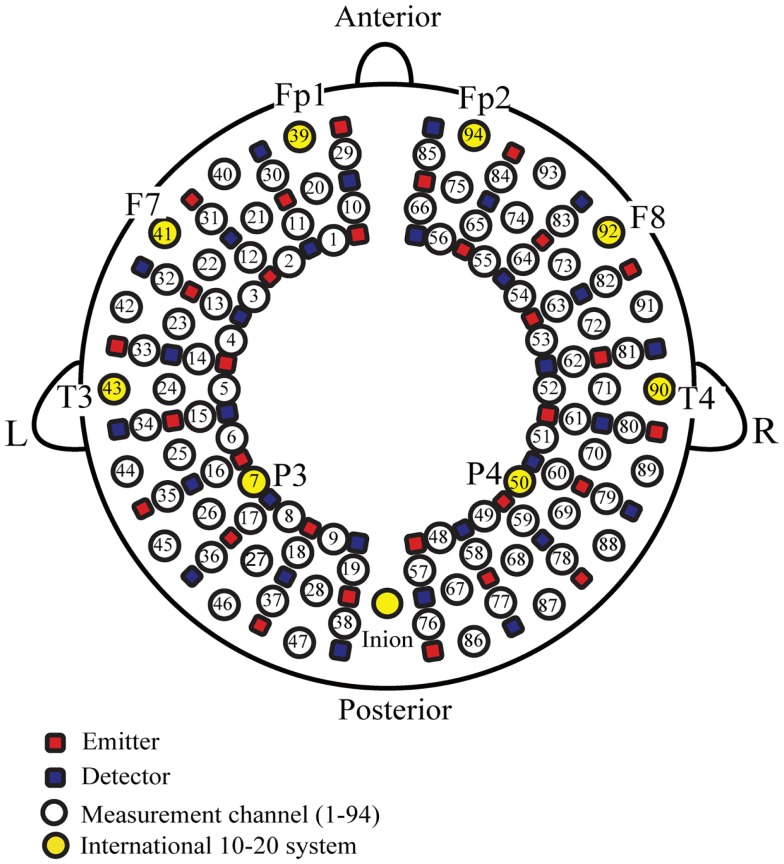
**Near-infrared spectroscopy probe and channel settings**. The lowest lines of the probes corresponded to the T3-Fp1-Fp2-T4 line according to the international 10–20 electrode system. The vertical midline of the channels was centered in the nasion – inion line and the channels between the fifth and sixth probe in the lowest line corresponded to T3 (channel 43) on the left and T4 (channel 90) on the right. L, left hemisphere, R, right hemisphere.

Fifteen incident and 15 detection probes arranged in a 3 × 10 grid (to form 47 channels) were fitted on each side of the head to form a total of 94 recording channels (Figure 1). The distance between emission and detection probes, which were developed for simultaneously measuring the frontal, temporal, parietal, and occipital regions in young infants, was approximately 2 cm. The very top of the parietal region was not covered with this probe, but our previous NIRS study using a probe array to cover the occipital, temporal, and centroparietal areas showed that auditory stimuli-induced oxy-Hb changes only occurred in the temporal area (Shibata et al., 2012). Previous NIRS studies have shown that the multichannel NIRS probe used in our study is highly sensitive for detecting cortical responses in response to various stimuli, including auditory stimuli in young infants (Homae et al., 2010; Taga et al., 2011; Watanabe et al., 2012). The 94 measurement channels were positioned at Fp1, Fp2, T3, T4, and inion locations in accordance with the international 10–20 electrode system that is used in electroencephalography (EEG) and that uses head circumference, the distance between the nasion and inion, and left and right preauricular points for positioning. The vertical midline of the channels was centered on the nasion–inion line and the channels between the fifth and sixth probe in the lowest line corresponded to T3 (channel 43) on the left and T4 (channel 90) on the right. According to the estimation of the cerebral location of the 10–20 electrode system placement in healthy adults (Homan et al., 1987; Okamoto et al., 2004), Fp1 (channel 39) and Fp2 (channel 94) roughly covered left and right superior and medial frontal gyri, Brodmann’s area 10. F7 (channel 41) and F8 (channel 92) roughly covered left and right inferior/middle frontal gyri, Brodmann’s areas 45 and 47, including Broca’s area. T3 (channel 43) and T4 (channel 90) likely covered left and right middle/superior temporal gyri, Brodmann’s areas 21 and 22, including Wernicke’s area. P3 (channel 7) and P4 (channel 50) likely covered left and right angular gyri and the supramarginal gyrus (Brodmann’s areas 39 and 40).

### Measurement protocol

Near-infrared spectroscopy recording was performed in a quiet room in the maternity unit. After measuring infant head size (head circumference, distance between nasion and inion, and left and right preauricular points), NIRS probes were positioned on the infant’s head.

Then, all infants performed an auditory block-design task. During a measurement session, a control period (pink noise) was administered at the beginning of each recording and each speech stimulation period (ADS or IDS) was alternated with a non-speech control period. The ADS and IDS conditions were repeated eight times using an additional control period at the end. Therefore, one NIRS measurement session comprised 16 stimulation periods (eight periods with the ADS and eight periods with the IDS) and 17 control periods. During each speech stimulation period, IDS stimuli or ADS stimuli read by one of four speakers were presented. Thus, the IDS and ADS stimuli read by four speakers were both presented twice. The order of the IDS and ADS stimuli was counterbalanced among the participants.

During the measurements, all infants were held by a pediatrician who supported the infants’ neck with three fingers (thumb, index, and middle fingers) so that the infants’ ears are not occluded and to keep the infants’ head upright. In addition, the sound speaker (Bose, Computer MusicMonitor 2.0 Speaker System) attached to a tripod with three flexible segmented legs (Gorillapod SLR-Zoom) was located approximately 30 cm in front of the infant’s head so the speaker to ear distance was equal for both ears. If infants moved during the measurements, the location of the speaker was modified to maintain an equal distance between each ear and the speaker. All stimuli were presented at a measured intensity of approximately 62 dB sound pressure level. The pediatrician wore headphones to prevent him from hearing the stimuli during the measurement. All measurements were recorded using a DV camera and a charge-coupled device (CCD) camera for later assessment of the participants’ body and facial movements. The total procedure took approximately 12 min.

### Data analysis

To examine the effect of preterm birth on functional cerebral development for speech processing, both cerebral activation and functional connectivity were assessed during the presentation of speech stimuli.

### Activation

Typically, concentration changes in oxy-Hb are more strongly correlated with BOLD signals than those in deoxy-Hb (Kato et al., 1993; Sakatani et al., 1999; Hoshi et al., 2000, 2001; Chen et al., 2002; Strangman et al., 2002, 2003; Jasdzewski et al., 2003; Taga et al., 2003). Therefore, our analysis mainly focused on hemodynamic changes reflected in oxy-Hb; however, deoxy-Hb was also analyzed. The oxy- and deoxy-Hb data were preprocessed using the plug-in-based analysis software Platform for Optical Topography Analysis Tools (Hitachi Ltd^1^) based on Matlab (The MathWorks, Inc.).

The raw data of oxy-Hb and deoxy-Hb from individual channels were digitally high-pass-filtered at 0.0133 Hz to remove components originating from task design and systematic fluctuations (Taga et al., 2003; Fuchino et al., 2006). A block was defined as the period between the 5 s before the onset of the speech stimulation period and 20 s after the end of the speech stimulation period. Blocks with motion artifacts (signal variations greater than two SDs of the mean over 0.2 s) were excluded. In preterm infants, the remaining blocks consisted of an average of 5.2 blocks per infant (SD = 0.2) for the ADS condition and 4.8 blocks (SD = 0.2) for the IDS condition (difference was not significant). In full-term infants, the remaining blocks consisted of an average of 5.4 blocks per infant (SD = 0.4) for the ADS condition and 5.1 blocks (SD = 0.5) for the IDS condition (difference was not significant). Channels with a low signal-to-noise ratio due to hair obstruction were also excluded. The mean number of channels remaining per infant was 92.8 (SD = 1.2) for the preterm group and 90.0 (SD = 3.9) for the full-term group (difference was not significant). Three infants in the full-term control group were excluded from further analysis because they had more than 20 eliminated channels (21%).

The oxy-Hb and deoxy-Hb of the remaining blocks were averaged and smoothed with a 5 s moving average. A baseline was linearly fitted between the means of the 5 s preceding the start of the speech stimulation period and 5 s of the 15–20 s period following the end of the speech stimulation period. The mean change in concentration of oxy-Hb and deoxy-Hb was measured for 10 s, beginning 5 s from the onset of each condition (IDS and ADS), and was calculated for each condition and for each channel.

Two-way mixed analysis of variance (ANOVA) was performed with repeated measures for the two measurement groups (full-term and preterm) as a between-subjects factor and the two stimuli conditions (IDS and ADS) as a within-subject factor for each channel. Effect sizes, as indexed by partial eta-squared ($\eta_{p}^{2}$), were also calculated.

### Functional connectivity

For each infant, pairwise correlation coefficients (*r*) were calculated between the time courses of oxy- or deoxy-Hb of all 94 measurement channels during the IDS and ADS conditions using the “corrcoef” Matlab function (The MathWorks Inc.). Then, Fisher’s *z*-transformation was applied to make the statistical distribution of correlation coefficients close to a normal distribution [*z* (*r*)]. Within the measurement groups (full-term and preterm), individual *z* (*r*) values were examined using one-sample *t*-test against zero in a measurement channel. The calculated *t* values were then converted to *z* statistics according to the formula $z=\left(t-\bar{t}\right)/\sigma$, where $\bar{t}$ and σ are the mean value and SD, respectively. We examined functional connectivity between representative regions of interest (ROIs) involved in speech processing that were located in the prefrontal (Fp1 and Fp2), frontotemporal (F7 and F8), temporal (T3 and T4), and temporoparietal (P3 and P4) regions.

To compare functional connectivity between the two groups (full-term and preterm) under two speech conditions (IDS and ADS), the *z* (*r*) values were subjected to a two-way mixed ANOVA with repeated measures for the two measurement groups (full-term and preterm) as a between-subjects factor and the two stimuli conditions (IDS and ADS) as a within-subject factor for the channels that exhibited significant functional connectivity for each ROI.

## Results

**Figure 2:**
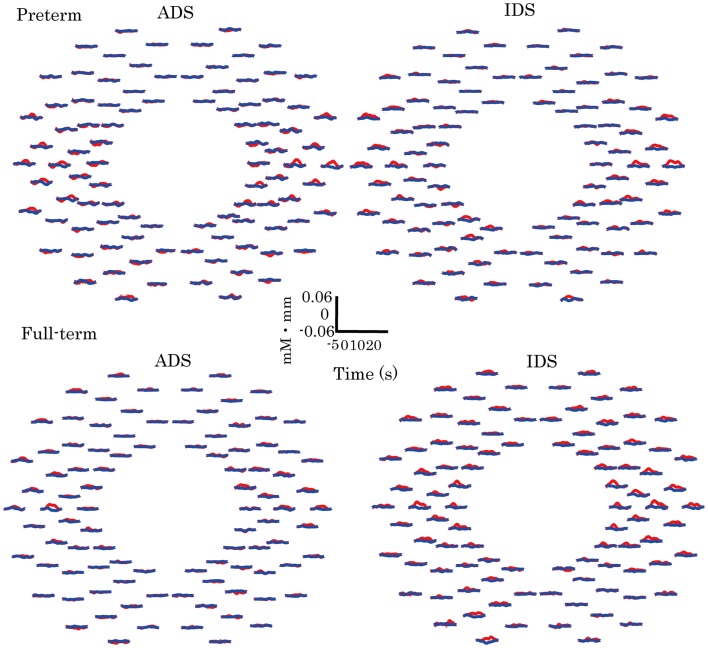
**Grand-averaged time courses of changes in oxy- and deoxy-Hb in ADS and IDS conditions for all 94 channels in the preterm and full-term infant groups**. Red and blue solid lines represent oxy-Hb and deoxy-Hb, respectively, in response to ADS and IDS conditions.

Hemodynamic responses in preterm and full-term infants to ADS and IDS compared with those to the control stimuli (pink noise) are shown in Figure 2. In the preterm and full-term groups, the channels in the bilateral temporal regions exhibited a distinctive increase in oxy-Hb in response to ADS and IDS stimuli.

### Activation

**Figure 3:**
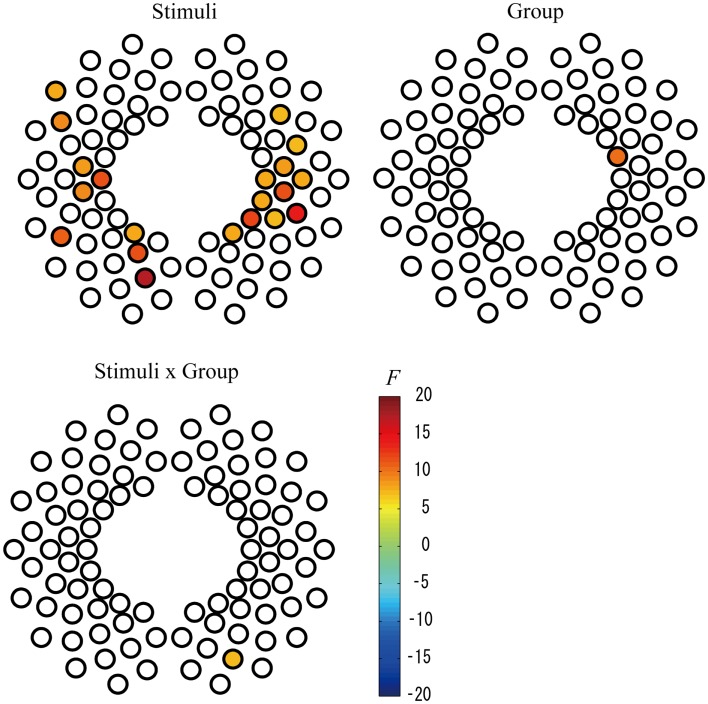
**Statistical maps (*F*-maps) for the main effect of stimuli (top left), the main effect of group (top right), and the interaction of stimuli and group (bottom left)**. The *F*-values for each channel are color-coded as indicated by the color bar (*p* < 0.01, uncorrected).

To examine the effect of preterm birth on cerebral activation in response to speech stimuli, we performed two-way mixed ANOVA for the two measurement groups (full-term and preterm) and two stimuli conditions (IDS and ADS) for each channel. We found that the magnitude of changes in the oxy-Hb response indicated a significant main effect of stimuli condition in large brain areas, including the bilateral frontotemporal, temporal, and temporoparietal regions, at the FDR-corrected *p* < 0.05 level (Table 2; Figure 3), and infants exhibited significantly larger responses to IDS than ADS. The same two-way ANOVA detected no significant main effect of group or interaction on the group and stimulus condition at the FDR-corrected *p* < 0.05 level. Using a more liberal *p* < 0.01 uncorrected for multiple comparisons, we detected a statistically significant main effect of group in Channel 53 (Ch 53) in the right frontotemporal area (Table 2; Figure 3), and full-term infants exhibited significantly larger responses than preterm infants. There was also a statistically significant interaction effect between group and stimulus condition in Ch 77 in the right occipital area at the uncorrected *p* < 0.01 level. The *post hoc* Bonferroni test revealed that full-term infants exhibited significantly larger responses than did preterm infants only under the ADS condition (*p* = 0.044).

**Table 2:** **Results of 2-way ANOVA of response magnitudes of changes in oxy-Hb**.

Channel		*F*	*P* (uncorrected)	*P* (FDR corrected)	$\eta_{p}^{2}$	
**MAIN EFFECT OF STIMULI**						
LH	5	11.93	0.0012	0.017	0.20	IDS > ADS
	8	8.24	0.0061	0.039	0.15	IDS > ADS
	14	8.84	0.0046	0.039	0.16	IDS > ADS
	15	9.77	0.0030	0.034	0.17	IDS > ADS
	18	12.16	0.0011	0.017	0.20	IDS > ADS
	28	18.05	0.0001	0.010	0.28	IDS > ADS
	32	9.60	0.0032	0.034	0.17	IDS > ADS
	35	11.73	0.0013	0.017	0.20	IDS > ADS
	41	8.20	0.0062	0.039	0.15	IDS > ADS
RH	44	7.94	0.0070	0.039	0.14	IDS > ADS
	50	12.84	0.0008	0.017	0.22	IDS > ADS
	51	8.04	0.0067	0.039	0.15	IDS > ADS
	52	8.46	0.0055	0.039	0.15	IDS > ADS
	60	7.58	0.0083	0.040	0.14	IDS > ADS
	61	12.35	0.0010	0.017	0.20	IDS > ADS
	62	8.95	0.0044	0.039	0.16	IDS > ADS
	70	14.65	0.0004	0.017	0.24	IDS > ADS
	71	8.05	0.0071	0.039	0.16	IDS > ADS
	72	7.58	0.0085	0.040	0.15	IDS > ADS
	73	7.53	0.0085	0.040	0.14	IDS > ADS
**MAIN EFFECT OF GROUP**						
RH	53	10.78	0.0019	0.180	0.18	Full > Preterm
**STIMULI × GROUP**						
RH	77	7.88	0.0075	0.45	0.16	Full > Preterm in ADS

*LH, left hemisphere, RH, right hemisphere*.

In the preterm group, we compared the activation patterns of seven ELBW infants (birth weight < 1000 g), 4 VLBW infants (birth weight of 1000 to <1500 g), and 14 infants with low birth weight (LBW; birth weight of 1500 to <2500 g). The analysis detected no significant main effect of birth weight or interaction on birth weight and stimulus condition at the FDR-corrected *p* < 0.05 level. Using the uncorrected *p* < 0.01, there was a statistically significant interaction effect of stimuli and group in the left temporal region [Ch 72, *F*(1,18) = 8.74, *p* = 0.0022]. The *post hoc* Bonferroni test showed that LBW infants exhibited significantly greater responses than ELBW infants, but only in the IDS condition (*p* = 0.010). In addition, preterm infants were divided into three groups on the basis of GA: extremely preterm infants (GA < 28 weeks); very preterm (GA between 28 and <32 weeks); moderate to late preterm (GA between 32 and <37 weeks). To investigate the effect of GA on these responses, we compared the activation patterns of 5 extremely preterm infants, 9 very preterm infants, and 11 moderate to late preterm infants. This analysis revealed no significant main effect of GA at birth or on the interaction between GA and stimulus condition at the FDR-corrected *p* < 0.05 and uncorrected *p* < 0.01 levels. Analyses of deoxy-Hb revealed no statistically significant differences in any comparisons at the uncorrected *p* < 0.01 level.

### Functional connectivity

**Figure 4:**
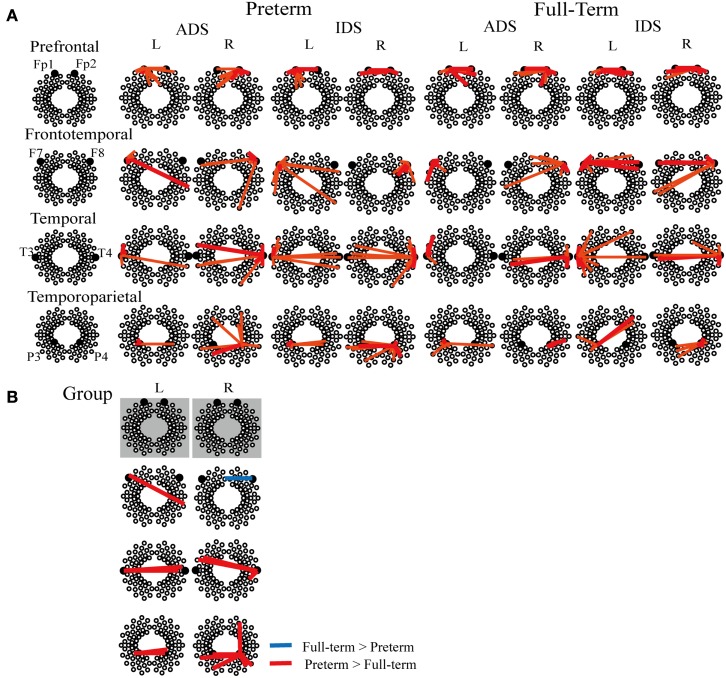
**(A)** Representative correlation maps corresponding to various measurement channels under ADS and IDS conditions in preterm and full-term neonates. The red thick lines represent a *z* threshold > 2.33 and the orange lines represent a *z* threshold > 1.65 (corresponding to *p* < 0.01 and *p* < 0.05, uncorrected for multiple comparisons, respectively). **(B)** Significant group differences in functional connectivity between preterm infants at term-equivalent age and full-term neonates (*p* < 0.05, FDR-corrected). Red and blue lines show increased connectivity in preterm infants and in full-term neonates, respectively. There was no significant group difference in functional connectivity between prefrontal ROIs (Fp1 and Fp2). L, left hemisphere, R, right hemisphere.

We evaluated the spatial distribution of functional connectivity in the selected ROIs located in the prefrontal (Fp1 and Fp2), frontotemporal (F7 and F8), temporal (T3 and T4), and temporoparietal (P3 and P4) regions (Figure 4A). Functional connectivity across multiple channels was observed both in the preterm and full-term groups.

Two-way mixed ANOVA of *z* (*r*) values for the two measurement groups (full-term and preterm) and two stimuli conditions (IDS and ADS) revealed a significant main effect of group at the FDR-corrected *p* < 0.05 level (Table 3; Figure 4B). The preterm group exhibited increased functional connectivity between the left frontotemporal and right temporal regions, between the left temporal and homotopic regions in the right hemisphere, between the left temporoparietal and homotopic regions in the right hemisphere, and between the right temporal and left frontotemporal regions. In addition, we found that the right temporoparietal region of preterm infants exhibited higher correlations with channels located in not only the left temporoparietal region but also the right prefrontal region, when compared that of full-term controls.

**Table 3:** **Results of 2-way ANOVA of *z* (*r*) values**.

ROI	Channel	*F*	*P* (uncorrected)	*P* (FDR corrected)	$\eta_{p}^{2}$	
**MAIN EFFECT OF GROUP**						
F7 (Ch41)	89	10.53	0.0022	0.0052	0.19	Preterm > full-term
F8 (Ch92)	10	10.05	0.0027	0.0055	0.17	Full-term > preterm
T3 (Ch43)	71	11.64	0.0015	0.0044	0.22	Preterm > full-term
	81	11.48	0.0014	0.0044	0.19	Preterm > full-term
T4 (Ch90)	32	8.97	0.0045	0.0087	0.17	Preterm > full-term
	42	10.73	0.0021	0.0052	0.20	Preterm > full-term
	71	12.67	0.0010	0.0044	0.24	Preterm > full-term
P3 (Ch7)	16	11.84	0.0013	0.0044	0.21	Preterm > full-term
	50	8.43	0.0058	0.0091	0.16	Preterm > full-term
	51	7.74	0.0079	0.012	0.15	Preterm > full-term
P4 (Ch50)	7	8.43	0.0058	0.0091	0.16	Preterm > full-term
	8	11.62	0.0014	0.0044	0.21	Preterm > full-term
	35	14.23	0.00048	0.0044	0.24	Preterm > full-term
	37	11.56	0.0014	0.0044	0.21	Preterm > full-term
	60	10.42	0.0023	0.0052	0.18	Preterm > full-term
	78	13.53	0.00063	0.0044	0.23	Preterm > full-term
	84	8.75	0.0049	0.0088	0.16	Preterm > full-term
	88	16.39	0.00021	0.0044	0.27	Preterm > full-term

However, the full-term group exhibited increased functional connectivity between the right frontotemporal and left prefrontal regions. There was no statistically significant main effect of stimulus condition or the interaction effect between group and stimulus condition at the FDR-corrected *p* < 0.05 and uncorrected *p* < 0.01 levels.

In the preterm group, we compared the functional connectivity of 7 ELBW infants, 4 VLBW infants, and 14 LBW infants. The analysis revealed no significant main effect of birth weight or the interaction between birth weight and stimulus condition at the FDR-corrected *p* < 0.05 level and uncorrected *p* < 0.01 levels. We also compared the functional connectivity of 5 extremely preterm infants, 9 very preterm infants, and 11 moderate to late preterm infants, and there was no significant main effect of GA at birth or the interaction between GA at birth and stimulus condition at the FDR-corrected *p* < 0.05 and uncorrected *p* < 0.01 levels.

### Correlation analysis

**Figure 5:**
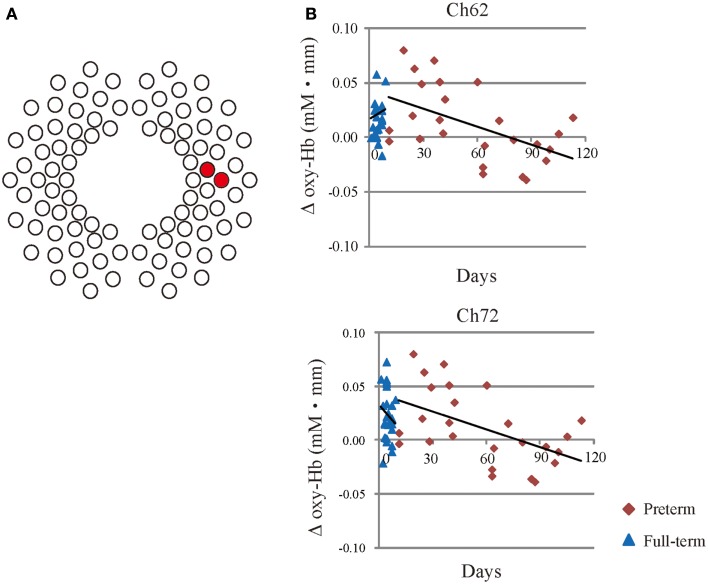
**(A)** The red circles represent channels with significant correlations between the PNA and oxy-Hb changes in response to IDS. **(B)** Scatter plots with regression lines show the relationship between oxy-Hb changes in Ch62 and Ch72 relative to PNA at the time of the scan in preterm (red rhombus) and full-term infants (blue triangles).

To examine the effect of PNA on cerebral activation and functional connectivity in response to speech, Pearson’s correlations were employed to examine the relationships between oxy-Hb changes or *z* (*r*) values in measurement channels that exhibited significant functional connectivity with each ROI and PNA at NIRS measurement. Oxy-Hb changes in response to IDS were inversely correlated with PNA in Ch62 and Ch72 in the right temporal region of the preterm group (Ch62: *r* = −0.43, *p* = 0.0021; Ch72: *r* = −0.39, *p* = 0.0077, see Figure 5). In addition, in the preterm group, oxy-Hb changes in response to ADS was inversely correlated with PNA in Ch32 in the left temporal region (*r* = −0.46, *p* = 0.00074).

With respect to functional connectivity during IDS and ADS presentation, functional connectivity during ADS presentation was inversely correlated with PNA between ROIs located in the left frontotemporal region (F7, Ch41) and Ch89 in the right temporal region in the preterm group (*r* = −0.62, *p* = 0.0010). There were no significant correlations between PNA and activation or functional connectivity in the full-term group.

## Discussion

In the present study, we examined cerebral activation and functional connectivity in response to IDS and ADS in full-term neonates and preterm infants at term-equivalent age using multichannel NIRS.

We found that IDS increased larger brain areas compared with ADS in full-term and preterm infants. This finding is consistent with behavioral results showing that full-term neonates have a behavioral preference for IDS (Cooper and Aslin, 1990). With respect to localization of activation, we provided evidence that human full-term neonates and preterm infants at term-equivalent age exhibit increased brain activation to IDS in the bilateral frontotemporal, temporal, and temporoparietal regions compared with ADS.

There are similarities in findings of responses to speech stimuli between our study and previous fMRI and NIRS studies in full-term neonates. Using fMRI, Perani et al. (2011) found significantly stronger responses were elicited in the bilateral temporal region and left inferior frontal region in full-term neonates when they were listening to normal IDS rather than when they were silent. In addition, using NIRS, Peña et al. (2003) demonstrated that increased temporal activation occurred in response to normal IDS compared with a silent baseline and IDS played backward. Furthermore, Sato et al. (2012) found larger hemodynamic responses in the left temporoparietal region, which is around the angular gyrus, during normal IDS compared with IDS played backward, but this difference was only observed when IDS was presented in the infants’ native language. Using multichannel NIRS covering the frontal, temporal, parietal, and occipital regions, the present study indicated that full-term neonates and preterm infants at term-equivalent age exhibited increased activation of brain regions known to be involved in speech processing, such as the frontotemporal, temporal, and temporoparietal regions, in response to IDS compared with ADS.

We compared the results with the full-term controls and the preterm infants exhibited decreased activity in the right temporal region in response to speech, although the significance was weak. Similar findings have been reported in preterm adolescents born with severe neonatal complications such as bronchopulmonary dysplasia. A previous MEG study demonstrated that preterm adolescents born under high-risk conditions exhibited decreased activation in the right superior temporal gyrus during auditory phonological tasks (Frye et al., 2010). In addition, a negative correlation was observed between cortical activity in response to IDS and PNA in the right temporal area (see Figure 5). This finding is consistent with previous ERP studies showing that altered neural response for processing speech sounds such as syllable or consonant changes occurs in preterm infants at term-equivalent age (Therien et al., 2004; Key et al., 2012). With the higher spatial resolution of multichannel NIRS, the present data supports these findings and suggests that increasing PNA in preterm infants was associated with decreased oxy-Hb changes with speech in the right temporal region, despite greater postnatal experience.

The group difference in cerebral activation between full-term and preterm infants was statistically weak but more obvious differences were found with respect to the cerebral functional connectivity. Preterm infants exhibited greater interhemispheric connectivity in the temporal and temporoparietal regions during speech processing compared with full-term controls. Previous MRI studies of brain functional connectivity have also shown that preterm children and adolescents exhibit different functional connectivity during processing language compared with full-term controls (Gozzo et al., 2009; Schafer et al., 2009; Myers et al., 2010). Increased connectivity between the left superior temporal gyrus (Wernicke’s area) and the right inferior frontal gyrus (Broca’s area homolog), and both the left and right supramarginal gyri, were observed in preterm children at school age. The functional connectivity between Wernicke’s area and the right supramarginal gyrus was increased in preterm adolescents (Myers et al., 2010). The present study demonstrated that preterm infants exhibited altered interhemispheric connectivity, particularly in the temporal and temporoparietal regions, when they reached the term-equivalent age.

Anatomical differences may have affected the development of cerebral activation and functional connectivity during speech processing in our study. Previous MRI studies and DTI studies have shown that the brain structure of preterm infants is different from that of term infants. At term, preterm infants exhibit reduced cortical sulcation, reduced white and gray matter volume, and alterations in their cerebral white matter microstructure (Huppi et al., 1998; Peterson et al., 2003; Inder et al., 2005; Kapellou et al., 2006; Woodward et al., 2006; Anjari et al., 2007; Srinivasan et al., 2007; Thompson et al., 2007; Aeby et al., 2009; Cheong et al., 2009; Skiold et al., 2010; Mento and Bisiacchi, 2012). In the present study, we did not compare the brain structures of preterm infants and full-term neonates, so it is possible that some preterm infants had subtle white matter injuries, which were undetectable using neonatal cranial ultrasonography and/or MRI.

In addition to intrauterine development, the altered cerebral activation and functional connectivity in response to speech in preterm infants compared with full-term infants may have been partly due to the different auditory experiences during their prenatal and postnatal periods. In our study, preterm infants had significantly lower GA at birth and higher PNA at NIRS measurement compared with full-term neonates. Therefore, at the time of measurement (at the term-equivalent age), preterm infants had fewer intrauterine sensory experiences and more extrauterine sensory experiences than full-term controls. The auditory system of the human fetus is functionally developed during the last trimester of pregnancy (Birnholz and Benacerraf, 1983; Richards et al., 1992; Hepper and Shahidullah, 1994; Eldredge and Salamy, 1996; Hall, 2000). External auditory stimuli are low-pass-filtered and attenuated by maternal tissues and fluids in the uterus; however, low-frequency speech and the maternal voice are preferentially transmitted to the fetus (Querleu et al., 1988, 1989; Benzaquen et al., 1990). A number of studies have shown that near-term fetuses can discriminate sounds frequently experienced *in utero*, such as the mother’s voice and maternal language, from unfamiliar sounds (Fifer and Moon, 1994; Kisilevsky et al., 2003, 2009; Smith et al., 2007). In addition, previous studies have demonstrated that human full-term neonates maintain this discriminative ability after birth (Decasper and Fifer, 1980; Ockleford et al., 1988; Fifer and Moon, 1994; Granier-Deferre et al., 2011). Preterm infants at term-equivalent age are likely to have less intrauterine auditory experience than full-term neonates. In fact, preterm infants at term exhibited no difference in ERP in response to the mother’s and strangers’ voices despite longer postnatal exposure to the maternal voice (Therien et al., 2004).

On the other hand, preterm infants at term-equivalent age are likely to have far more experience with the extrauterine environment. The mean PNA at NIRS measurement was approximately 56 days, and most preterm infants are treated in NICU during this period. In NICU, infants born preterm are likely to be exposed to intense noise levels above 50 dB (A), which exceed recommended levels (Lasky and Williams, 2009), and higher frequency noise than that experienced *in utero* (Livera et al., 2008). Preterm infants treated in NICU seem to be sensitive to the nosocomial auditory environment and exhibited decrease in respiratory rates and blood oxygen saturations in response to 5–10 dB sounds (A) above background noise levels (Kuhn et al., 2012). These postnatal experiences may also influence development of brain function in response to speech in preterm infants.

Previous structural and functional studies have suggested that intrauterine rather than extrauterine development appears to have an adequate impact on brain maturation in preterm infants and that brain maturation appears to constrain the effects of postnatal experience on speech discrimination during the early stages of language acquisition in preterm infants. (Peña et al., 2010; Mento and Bisiacchi, 2012). Furthermore, in the absence of a sufficiently long period of intrauterine development, extrauterine stimulation may have negative effects on brain maturation in some infants born preterm (Huppi et al., 1996; Als et al., 2004). Our results showed that oxy-Hb changes in response to IDS were inversely correlated to PNA in the right temporal region only in the preterm group.

In conclusion, the present study demonstrated that preterm infants at term exhibited decreased activation in the right temporal region and increased connectivity between the bilateral temporal and bilateral temporoparietal regions during speech presentation when compared with full-term neonates. This difference might suggest that preterm infants and full-term neonates follow different developmental trajectories during the postnatal period. It is also possible that early neural alternations in the development of speech perception may be related to later difficulties with language development in preterm children and adolescents. Further studies are required to explore the relationship between hemodynamic responses to speech at term and later development.

## Conflict of Interest Statement

The authors declare that the research was conducted in the absence of any commercial or financial relationships that could be construed as a potential conflict of interest.

## Footnotes

^1^http://goo.gl/4Nqgo
